# Modeling dynamic velocity-based gestational weight gain trajectories in advanced maternal age: association with perinatal outcomes

**DOI:** 10.3389/fmed.2026.1782650

**Published:** 2026-09-09

**Authors:** Guobin Li, Qingmei Lin, Jiamiao Wu, Jingxue Feng, Yingying Song, Lujia Li, Feng Tan, Pinmo Wu, Huanchang Yan, Fangfang Zheng, Xiu Qiu, Xiaobo Guo, Yu Liu

**Affiliations:** 1 Department of Statistical Science, School of Mathematics, Sun Yat-Sen University, Guangzhou, China; 2 Foshan Women and Children Hospital Affiliated to Guangdong Medical University, Foshan, China; 3 The Born in Guangzhou Cohort Study Group, Guangzhou Women and Children's Medical Center, Guangzhou Medical University, Guangzhou, China; 4 Division of Women Health Care, Guangzhou Women and Children's Medical Center, Guangzhou Medical University, Guangzhou, China; 5 School of Public Health and Management, Guangzhou University of Chinese Medicine, Guangzhou, China; 6 Department of Epidemiology and Biostatistics, School of Public Health, Tongji Medical College, Huazhong University of Science and Technology, Wuhan, Hubei, China; 7 Department of Medical Statistics, School of Public Health, Guangzhou University of Chinese Medicine, Guangzhou, China; 8 School of Traditional Chinese Medicine Healthcare, Guangdong Food and Drug Vocational College, Guangzhou, China

**Keywords:** advanced maternal age women, gestational weight gain trajectories, latent class mixed models, perinatal outcomes, velocity dynamics

## Abstract

**Background:**

Static gestational weight gain (GWG) guidelines may be inadequate for women of advanced maternal age (AMA), a population with distinct physiological characteristics including diminished metabolic flexibility and elevated baseline risks. This study seeks to delineate dynamic GWG trajectories in this cohort to facilitate more precise risk stratification during pregnancy.

**Methods:**

This retrospective cohort study analyzed routinely collected antenatal weight measurements from 3,818 AMA women. Distinct GWG trajectory patterns across the second and third trimesters were identified using latent class mixed models, from which velocity dynamics were derived as first derivatives and assessed for associations with key perinatal outcomes.

**Results:**

Five GWG trajectory patterns were identified: low-gain with terminal acceleration (LGT-Accel, 10.6%), steady-gain with mid-gestation deceleration (SGM-Decel, 29.7%, reference), modest-gain with terminal surge (MGT-Surge, 21.6%), high-gain with trajectory attenuation (HGT-Atten, 26.6%), and maximal-gain with steepest slope (MGS-Slope, 11.6%). All followed a consistent four-phase kinetic profile but differed in cumulative gain and velocity transitions. Compared to the SGM-Decel group, the LGT-Accel and MGT-Surge groups increased risks of low birthweight and small for gestational age, whereas the HGT-Atten and MGS-Slope groups were associated with macrosomia and large for gestational age, with the MGS-Slope group also linked to low birthweight risk.

**Conclusion:**

This study reveals that GWG in AMA women follows heterogeneous dynamic trajectories that are not captured by static, one-size-fits-all guidelines. Moving beyond total gain to monitor phase-specific velocity dynamics offers a promising paradigm for enhancing real-time risk assessment and enabling personalized antenatal care in this vulnerable population.

## 1 Introduction

Gestational weight gain (GWG) represents a critical dynamic adaptation of the feto-maternal unit (1), with trajectory patterns significantly influencing perinatal outcomes (2–5). Current guidelines, such as those from the Chinese Nutrition Society (CNS) (6) and the US Institute of Medicine (7), primarily assess GWG using total gain or interval-averaged weekly gain, which do not fully capture changes in the pace of weight gain within gestational intervals. By modeling longitudinal trajectories and time-varying velocity, our approach identifies when GWG accelerates or decelerates, providing complementary temporal information that may distinguish women with similar conventional GWG measures but different dynamic patterns.

Advanced maternal age (AMA, ≥35 years at delivery) constitutes a growing demographic in China (8, 9), associated with elevated risks of adverse maternal-fetal outcomes (10–13). These risks are largely attributed to age-related biological factors, including altered metabolic adaptation, vascular changes, and placental dysfunction, rather than to gestational weight gain alone. Nevertheless, GWG remains one of the few modifiable and routinely monitored indicators throughout pregnancy. Despite its widespread use in clinical practice, evidence characterizing longitudinal GWG trajectories and their temporal dynamics specifically among AMA pregnancies remains limited. Reliance on aggregate metrics, such as total GWG or mean weight gain velocity, may obscure important temporal heterogeneity that could be relevant for identifying fetal growth–related risks.

Emerging research underscores the importance of trajectory kinetics (14–16). Velocity patterns, reflecting the rate and timing of weight accrual, may offer deeper physiological insights than aggregate measures. Nevertheless, comprehensive analyses of GWG trajectories and their velocity profiles in high-risk AMA populations are lacking, limiting the ability to evaluate their potential utility for antenatal risk stratification.

To address these gaps, we conducted a retrospective cohort study utilizing clinical data from Foshan Women and Children Hospital in Foshan, China. To ensure data quality and reduce potential bias arising from longitudinal data irregularities, a conditional growth percentile approach was applied for data cleaning. Subsequently, latent class mixed-effects models (LCMMs) were employed to identify distinct GWG trajectory patterns across the 2nd and 3rd trimesters. The velocity dynamics of weight gain were further derived by calculating the first derivatives of these trajectory functions. By characterizing GWG patterns and their temporal dynamics in AMA pregnancies, this study aims to provide clinically relevant insights for early risk stratification and monitoring, rather than to replace existing guideline-based management.

## 2 Methods

### 2.1 Study population

**Figure 1:**
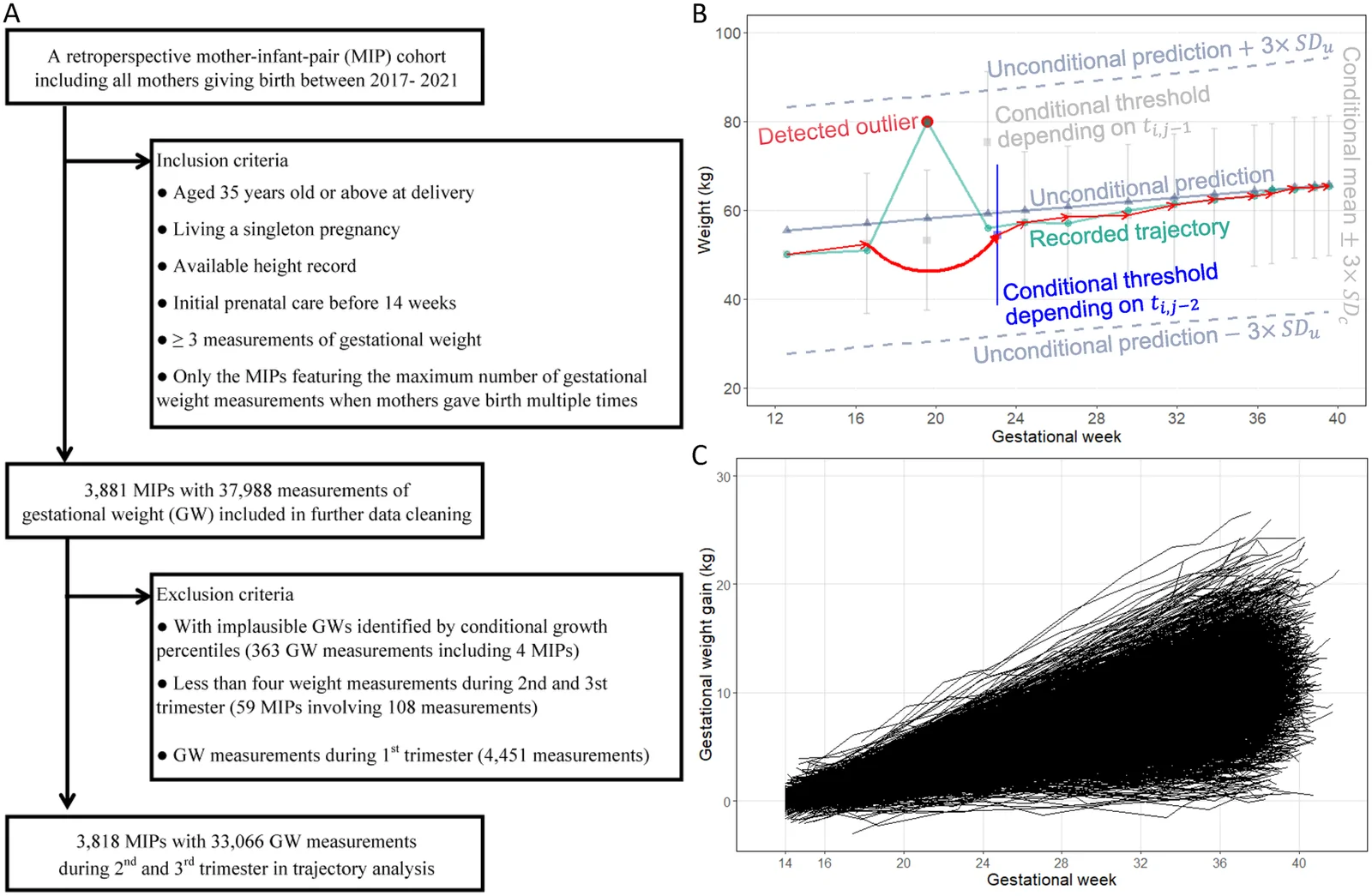
Data cleaning process and gestational weight data preparation. **(A)** Flowchart of inclusion and exclusion criteria for the mother–infant-pair (MIP) cohort. **(B)** Example of implausible measurement detection using conditional growth percentiles. The grey line shows the threshold based on the previous measurement; The blue line indicates the threshold recalculated when the previous point is outlier; red points mark outliers. **(C)** Individual trajectories of gestational weight gain (GWG) across gestational weeks after data cleaning.

Between 2017 and 2021, a total of 35,840 deliveries occurred at Foshan Women and Children Hospital. From this source population, mother–infant pairs involving women aged 35 years or older at delivery were identified through the hospital health information system and screened for eligibility. Additionally, MIPs were deemed eligible only if mothers had recorded height data, initiated antenatal care before 14 weeks of gestation, and underwent ≥3 antenatal weight measurements throughout pregnancy until delivery (16). In cases where a mother gave birth multiple times at an advanced maternal age, only the MIPs featuring the maximum number of gestational weight measurements were retained. Consequently, 3,881 MIPs with 37,988 gestational weight measurements were included in this study (Figure 1A).

This study was approved by the Ethics Committee of Foshan Women and Children Hospital (No.: FSFY-MEC-2024-060). Data used in this study were anonymous and no individual identifiable information was available for the analysis.

### 2.2 Quality control for gestational weight trajectory

The temporal pattern of serial gestational weight measurements indicated consistent adherence to antenatal care schedules among pregnant women (Supplementary Figure 1A), but substantial heterogeneity in frequency were observed (Supplementary Figure 1B). These weight measurements contained a substantial number of implausible values. (Supplementary Figure 1C), as reported by previous study (17). Given the longitudinal dependence inherent in repeated weight measurements, we employed the conditional growth quantile approach (18) to clean gestational weight trajectories.

To calculate conditional growth percentiles, a mixed effect model is first built to describe the repeated weight measurements $W_{ij}$ of individual *i* at occasion *j* as a function of gestational age $t_{ij}$ (18):

$$W_{ij}=\alpha_{0i}+\alpha_{1i}t_{ij}+\omega_{ij},$$

where $\alpha_{0i}$ and $\alpha_{1i}$ are individual-specific coefficients, which have fixed ($\alpha_{0}$ and $\alpha_{1}$) and random ($u_{0i}$ and $u_{1i}$) components, with

$$\alpha_{0i}=\alpha_{0}+u_{0i}\;\mathrm{and}\;\alpha_{1i}=\alpha_{1}+u_{1i}.$$

The fixed coefficients $\alpha_{0}$ and $\alpha_{1}$ are shared by all individuals, while the error terms $u_{i}=(u_{0i},u_{1i})$ are unobserved random variables that capture the individual departures from the population average trajectory, $\alpha_{0}+\alpha_{1}t_{ij}$. The $\omega_{ij}s$ capture the distance between the observed data for the *i*^th^ individual to the true individual-specific trajectory, $\alpha_{0i}+\alpha_{1i}t_{ij}$ (19). The parameter estimation of the model is used to derive the conditional mean weight and a ± 3 standard deviation (SD) range for an individual's weight at time $t_{ij}$, given their weight at time $t_{i,j-1}$. Weight measurements which are below −3 SD or above +3 SD are classified as outliers. Critically, the plausibility assessment of post-outlier observations utilize pre-outlier data, since the conditional mean calculation exhibits strong dependence on prior measurements (Figure 1B). Eventually, conditional growth percentiles identified 363 implausible values, enhancing both biological plausibility and continuity in gestational weight trajectory data (Supplementary Figure 1D).

Then, we eliminated MIPs with fewer than four antenatal weight measurements between the 2nd and 3rd trimesters. Finally, 3,818 MIPs with 33,066 gestational weight measurements were involved for GWG analysis.

### 2.3 Assessment of weight gain trajectory and velocity

In this study, pre-pregnancy BMI was calculated as “weight (kg)/[height (m)]^2^” at the first antenatal care visit, and was further divided into underweight (<18.50), normal weight (18.50–23.99), overweight (24.00–29.99), and obese (≥30.00) (20). Maternal GWG in the 2nd and 3rd trimesters (from 14 weeks to delivery) was defined as the difference between weight measured at each antenatal examination and the weight recorded at the 13th week (16). Due to lack of weight measurements exactly at the 13th week (Supplementary Figure 2A), they were imputed by prediction using the aforementioned mixed effect model. Such prediction was assessed using weight measurements in the 1st trimester, which indicated a justifiable range of predictive error (Supplementary Figure 2B). Subsequently, total GWG during pregnancy was classified as insufficient, adequate, or excessive, according to the 2021 CNS guideline (6).

Latent class mixed model (LCMM) (21) is used to investigate GWG trajectory patterns of the 2nd and 3rd trimesters. The GWG $y_{ij|g}$ of individual *i* at occasion *j* in class *g* was specified as a class-specific natural cubic spline function $f_{g}$ of gestational age $t_{ij}$:

$$y_{ij|g}(t_{ij})=f_{g}(t_{ij})+\beta_{0i|g}+\beta_{1i|g}t_{ij}+\varepsilon_{ij}.$$

The $f_{g}$ employs a B-spline basis that ensure twice continuously differentiability, and this basis depends on a set of interior knots and boundary knots in the range of $\left\{t_{ij}\right\}$ (22). It has the form of

$$f_{g}(t_{ij})=\overset{df}{\underset{k=0}{\sum }}\;\gamma_{k|g}B_{k}(t_{ij},3),$$

where $B_{k}(t_{ij},3)$ s are B-spline basis functions with degree 3 (23) and df is the degree of freedom typically chosen as the number of knots plus 1 (24). Here, $\gamma_{k|g}$ denotes the coefficient associated with the *k*-th B-spline basis function for class g. The random effects $\beta_{0i|g}$ and $\beta_{1i|g}$ are introduced to model between-subject variation in initial GWG and rate of change at time $t_{ij}$, where $\left(\beta_{0i|g},\beta_{1i|g}{)}^{T}∼N(0,\Sigma \right)$ and the covariance matrix Σ is diagonal. Residual errors $\varepsilon_{ij}$ are assumed to be independent and identically distributed as $N(0,\sigma_{\varepsilon }^{2})$, reflecting the differences between observed and predicted values. Maximum likelihood estimators for the parameters are obtained using a modified Marquardt algorithm in R lcmm package (21).

To determine the best model fit, we explored latent class numbers from 1 to 7 and the natural cubic spline basis with 3 to 10 degrees of freedom which chose knots at suitably chosen quantiles (24). The optimal model was selected according to the following criteria (25–27): (1) significant improvement of the model in Bayesian Information Criterion (BIC, Supplementary Figure 3A); (2) no less than 314 (>10%) participants in any single trajectory group (Supplementary Figure 3B); (3) high mean posterior probabilities (>0.7, Supplementary Figure 3C); (4) relatively high proportion (>0.65) of participants with posterior probabilities >0.7 in each trajectory group (Supplementary Figure 3D); (5) clinical interpretability.

The first derivative of expression profiles in the trajectory modelling records the velocity of GWG over time. The derivative of the mean population curve $f_{g}$ for class *g* is

$$f_{g}^{{}^{\prime }}(t_{ij})=\overset{df}{\underset{k=0}{\sum }}\;{\hat{\gamma }}_{k|g}B_{k}^{{}^{\prime }}(t_{ij},3).$$

where $B_{k}^{\prime }(t_{ij},3)s$ are generated using the “predict()” command with the argument “deriv = 1” from the R splines package. Here, ${\hat{\gamma }}_{k|g}$ denotes the estimates of $\gamma_{k|g}.$

### 2.4 Statistical analysis

Continuous variables are presented as means ± standard deviations (SDs), and categorical variables as frequencies (percentages). Between-group comparisons for continuous variables were performed using analysis of variance (ANOVA) or the Kruskal–Wallis test, as appropriate. Categorical variables were compared using the Chi-square or Fisher's exact test.

To evaluate the associations between gestational weight gain (GWG) trajectory groups and antenatal outcomes, including preterm birth (PTB), low birth weight (LBW), macrosomia, small for gestational age (SGA), and large for gestational age (LGA), we constructed a multivariable logistic regression model adjusted for maternal age, pre-pregnancy BMI, conception mode, delivery mode, parity, and fetal sex. To assess the influence of pregnancy-related clinical conditions, we conducted a sensitivity analysis in which the primary model was additionally adjusted for gestational diabetes mellitus and hypertensive disorders of pregnancy.

To examine whether the identified GWG patterns were consistent across parity groups, we conducted an additional parity-stratified descriptive analysis. Using the posterior class assignments obtained from the primary latent class mixed model, the class-specific GWG trajectory models were refitted separately for primiparous and multiparous women, and the corresponding velocity curves were derived as the first derivatives of the fitted trajectories.

All analyses were conducted using R statistical software (version 4.4.2). Statistical tests were two-sided, with *p* < 0.05 considered statistically significant.

## 3 Results

### 3.1 Cohort setup and its characteristics

Between 2017 to 2021, 3,818 MIPs meeting the inclusion criteria of AMA, singleton live birth, and ≥3 antenatal weight measurements during pregnancy were identified through Foshan Women and Children Hospital information system (Figure 1A). Subsequently, the conditional growth percentile approach using ±3 SD from the conditional mean as the criterion to access observation plausibility within individual trajectories (Figure 1B) detected 363 implausible values among 37,988 weight measurements. Following data imputation for gestational weight at the 13th week and data sufficiency assessment, 3,818 MIPs with 33,066 weight measurements were included for GWG trajectory reconstruction during the 2nd and 3rd trimester (Figure 1D).

Among 3,818 AMA mothers, the mean delivery age was 38.0 ± 2.4 years; 753 (19.7%) were of super-advanced maternal age (≥40 years). A total of 3,354 (87.8%) conceived naturally, 3,163 (82.8%) were multiparous, and 2,485 (65.1%) delivered by cesarean. Regarding BMI categories: 222 (5.8%) were underweight, 2,420 (63.4%) were normal weight, 926 (24.3%) were overweight, and 250 (6.6%) were obese. Further participant characteristics are detailed in Supplementary Table 1.

### 3.2 Frequent deviations from recommended GWG criteria

**Figure 2:**
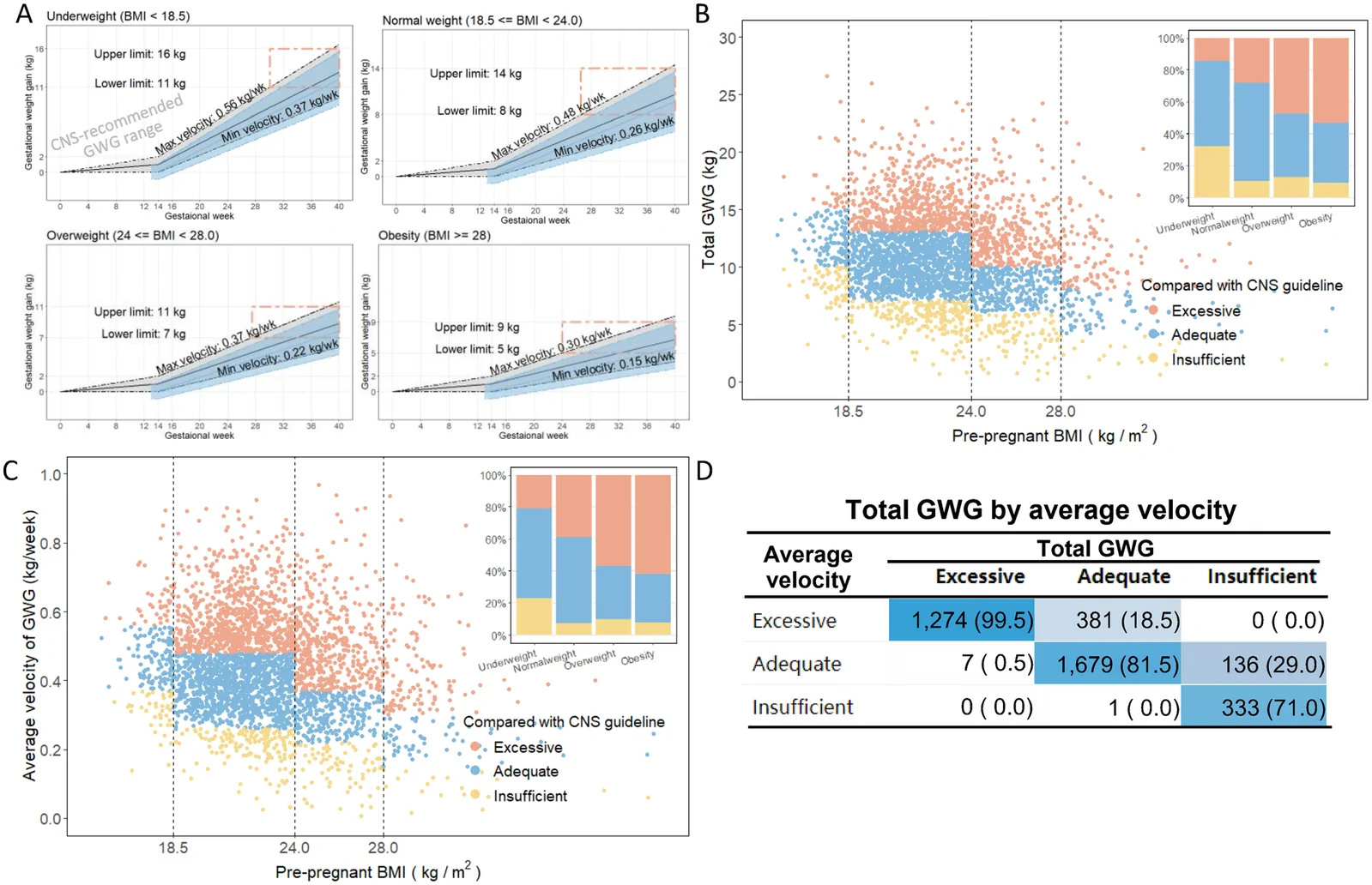
Gestational weight gain compared with Chinese nutrition society (CNS) guidelines across pre-pregnancy BMI groups. **(A)** Recommended GWG ranges (upper and lower limits) by pre-pregnancy BMI category (underweight, normal weight, overweight, obesity). **(B)** Total GWG by pre-pregnant BMI category. Data are categorized by CNS recommendations as inadequate (below), adequate (within), or excessive (above) the recommended range. The inset bar plot shows the proportion of each GWG category by BMI group. **(C)** Average GWG velocity (kg/week) by pre-pregnant BMI category. Data are categorized by CNS recommendations as inadequate (below), adequate (within), or excessive (above) the recommended range. The inset bar plot shows the proportion of each GWG velocity category by BMI group. **(D)** Cross-tabulation of GWG adequacy classified by total GWG (columns) and average velocity (rows). Values are shown as counts (percentages).

According to the CNS guidelines, achieving term delivery (≥36 weeks) solely by adhering to the minimum average GWG rate may result in falling below the lower threshold for the recommended range (Figure 2A). Consequently, maintaining a weight gain rate above the median of the CNS-recommended range during mid-to-late pregnancy is likely necessary for pregnant individuals to meet the total GWG targets. Notably, we established reference curves for mid-late pregnancy GWG progression relative to weight gain at 13 weeks, serving as comparison benchmarks (Figure 2A and Supplementary Figures 4A–D).

Among AMA women, the proportion achieving total GWG within CNS guidelines declined across increasing pre-pregnancy BMI categories, whereas the proportion exceeding the guideline limits increased (Figure 2B). In contrast, those with insufficient total GWG represented a minority. A parallel trend was observed for deviations in average weight gain velocity (Figure 2C).

Strikingly, 29.0% of AMA women with insufficient total GWG nonetheless exhibited adequate weight gain velocity. Conversely, among those achieving adequate total GWG, 18.5% demonstrated velocities exceeding guideline recommendations (Figure 2D). This discordance indicates that cumulative gain and interval-averaged velocity capture different aspects of GWG, but neither fully characterizes the underlying temporal pattern.

### 3.3 GWG trajectory patterns and their associations with birth outcomes

**Figure 3:**
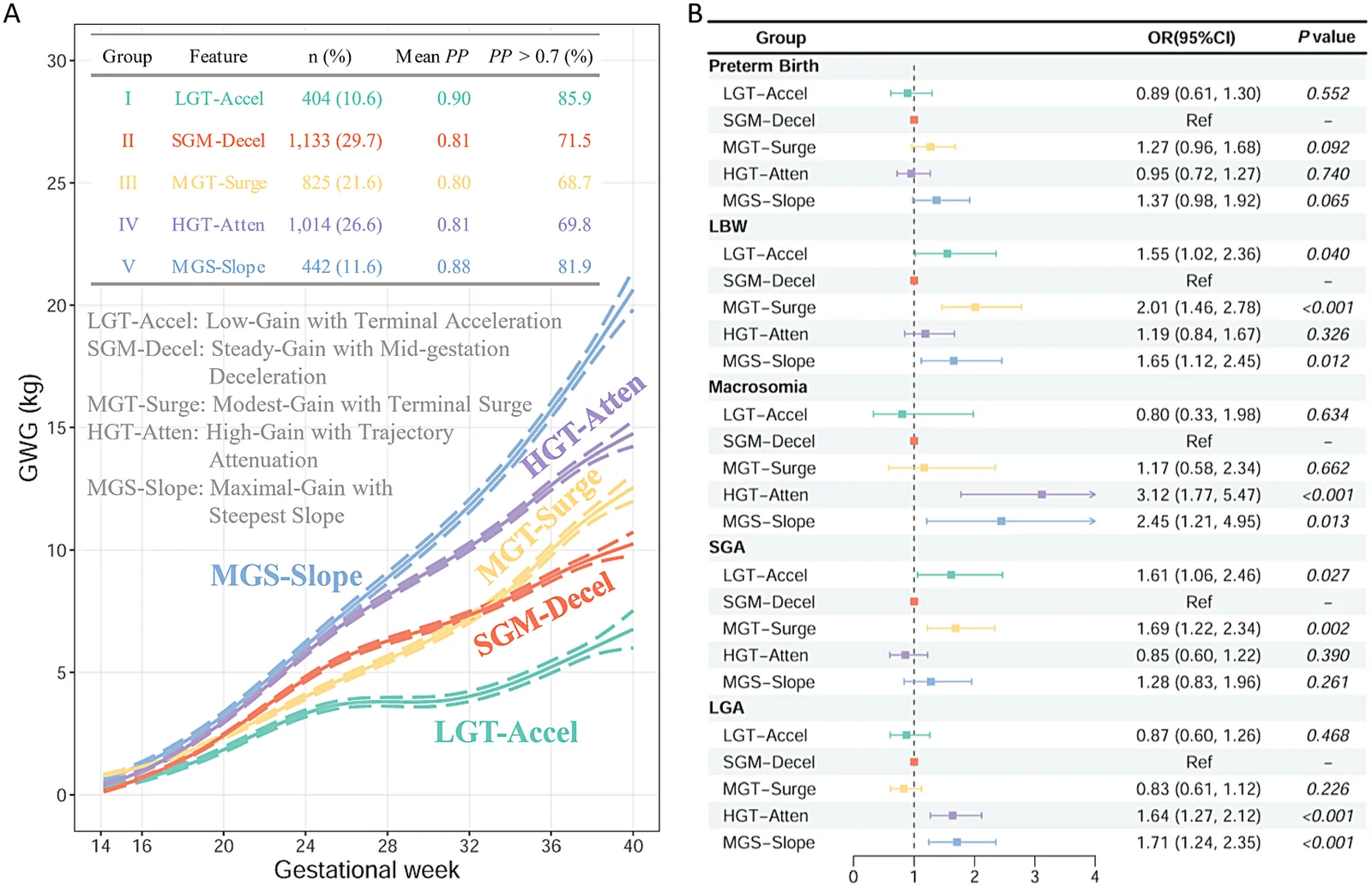
Gestational weight gain (GWG) trajectories and associations with pregnancy outcomes. **(A)** GWG trajectories were identified using a latent class mixed model (LCMM). The inset table shows the number of participants, mean posterior probability (Mean PP), and the proportion with *PP* > 0.7 for each group. **(B)** Associations between GWG trajectory groups and pregnancy outcomes. Models adjusted for maternal age, pre-pregnancy BMI, conception mode, delivery mode, parity, and fetal sex.

The LCMM identified five distinct GWG trajectory patterns in AMA women (Figure 3A and Supplementary Figure 5), with the optimal number of classes and spline degrees of freedom (see Material and Methods). Group I (*n* = 404, 10.6%) exhibits the lowest cumulative GWG (≈6.8 kg at term), characterized by a plateau phase during late second to early third trimester followed by marked end-gestational acceleration (mean posterior probability: 0.90; >0.7 probability: 85.9%), designated as Low-Gain with Terminal Acceleration (LGT-Accel). Group II (*n* = 1,133, 29.7%) demonstrates subminimal cumulative gain (≈10.3 kg at term), differentiated by decelerated growth velocity at second-trimester completion (mean posterior probability: 0.81; >0.7 probability: 71.5%) and termed Steady-Gain with Mid-Gestation Deceleration (SGM-Decel). Group III (*n* = 825, 21.6%) maintains modest cumulative gain (≈12.6 kg at term), featuring steady progression with a growth surge initiating at third-trimester onset (mean posterior probability: 0.80; >0.7 probability: 68.7%), classified as Modest-Gain with Terminal Surge (MGT-Surge). Group IV (*n* = 1,014, 26.6%) presents high cumulative gain (≈14.7 kg at term), showing trajectory mirroring Group V until 25 weeks with subsequent attenuation (mean posterior probability: 0.81; >0.7 probability: 69.8%), defined as High-Gain with Trajectory Attenuation (HGT-Atten). Group V (*n* = 442, 11.6%) manifests maximal cumulative gain (≈20.6 kg at term) with the steepest sustained slope commencing at 14 weeks (mean posterior probability: 0.88; >0.7 probability: 81.9%), notated as Maximal-Gain with Steepest Slope (MGS-Slope).

Despite statistically significant inter-group differences, the mean maternal age at delivery across all groups was approximately 38 years, with a mean gestation age of around 38 weeks (Supplementary Table 2). Specifically, the LGT-Accel group exhibited the oldest maternal age (38.5 ± 2.7 years), the highest proportion of super-AMA pregnancies (29.5%), the maximum pre-pregnancy BMI (25.1 ± 3.6 kg/m^2^), a high cesarean section rate (64.9%), and the lowest average total GWG (5.2 ± 1.8). Conversely, the MGS-Slope group had the youngest maternal age (37.5 ± 2.1 years), the lowest proportion of super-AMA pregnancies (13.3%), the highest cesarean section rate (71.9%), and the highest average total GWG (16.3 ± 3.1). Among these groups, the SGM-Decel group was designated as the reference group for comparative analysis due to its favorable obstetric outcomes: moderate average total GWG (8.9 ± 1.7 kg), second-lowest preterm birth rate (10.4%), highest proportion of infants with normal birth weight (92.1%), and highest proportion of AGA infants (82.3%).

Using the SGM-Decel group as the reference, multivariable logistic regression models were established to evaluate associations between GWG trajectory groups and obstetric outcomes (Figure 3B). Specifically, the LGT-Accel group exhibited significantly elevated risks of LBW (OR 1.55, 95% CI: 1.02–2.36; *P* = 0.040) and SGA (1.61, 1.06–2.46; 0.027), consistent with fetal growth restriction mechanisms. Conversely, this group demonstrated non-significant associations with LGA (0.87, 0.60–1.26; 0.468) and macrosomia (0.80, 0.33–1.98; 0.634), suggesting that selective metabolic dysregulation primarily impacts undergrowth phenotypes. Similarly, the MGT-Surge group showed the most pronounced risk for LBW (2.01, 1.46–2.78; <0.001) and SGA risk (1.69, 1.22–2.34; 0.002) among all groups, along with a non-significant trend toward PTB (1.27, 0.96–1.68; 0.092). No statistically significant associations were detected with LGA (0.83, 0.61–1.12; 0.226) or macrosomia (*P* = 0.662).

Furthermore, in the primary analysis, the HGT-Atten group presented the highest risk of macrosomia (3.12, 1.77–5.47; <0.001) and significantly elevated LGA risk (1.64, 1.27–2.12; <0.001). No statistically significant associations were observed with LBW or SGA; therefore, the outcome profile of this trajectory was primarily characterized by fetal overgrowth. Finally, in the primary analysis, the MGS-Slope group was associated with significantly increased risks of LGA (1.71, 1.24–2.35; <0.001), macrosomia (2.45, 1.21–4.95; 0.013), and LBW (1.65, 1.12–2.45; 0.012). The associations with PTB (1.37, 0.98–1.92; 0.065) and SGA (1.28, 0.83–1.96;0.261) were not statistically significant.

Sensitivity analyses additionally adjusting for gestational diabetes and hypertensive disorders confirmed the main findings, with the exception of the MGS-Slope-LBW and LGT-Accel-LBW associations (relative to SGM-Decel), which lost statistical significance (Supplementary Table 3).

### 3.4 GWG velocity patterns during the 2nd and 3rd trimesters

**Figure 4:**
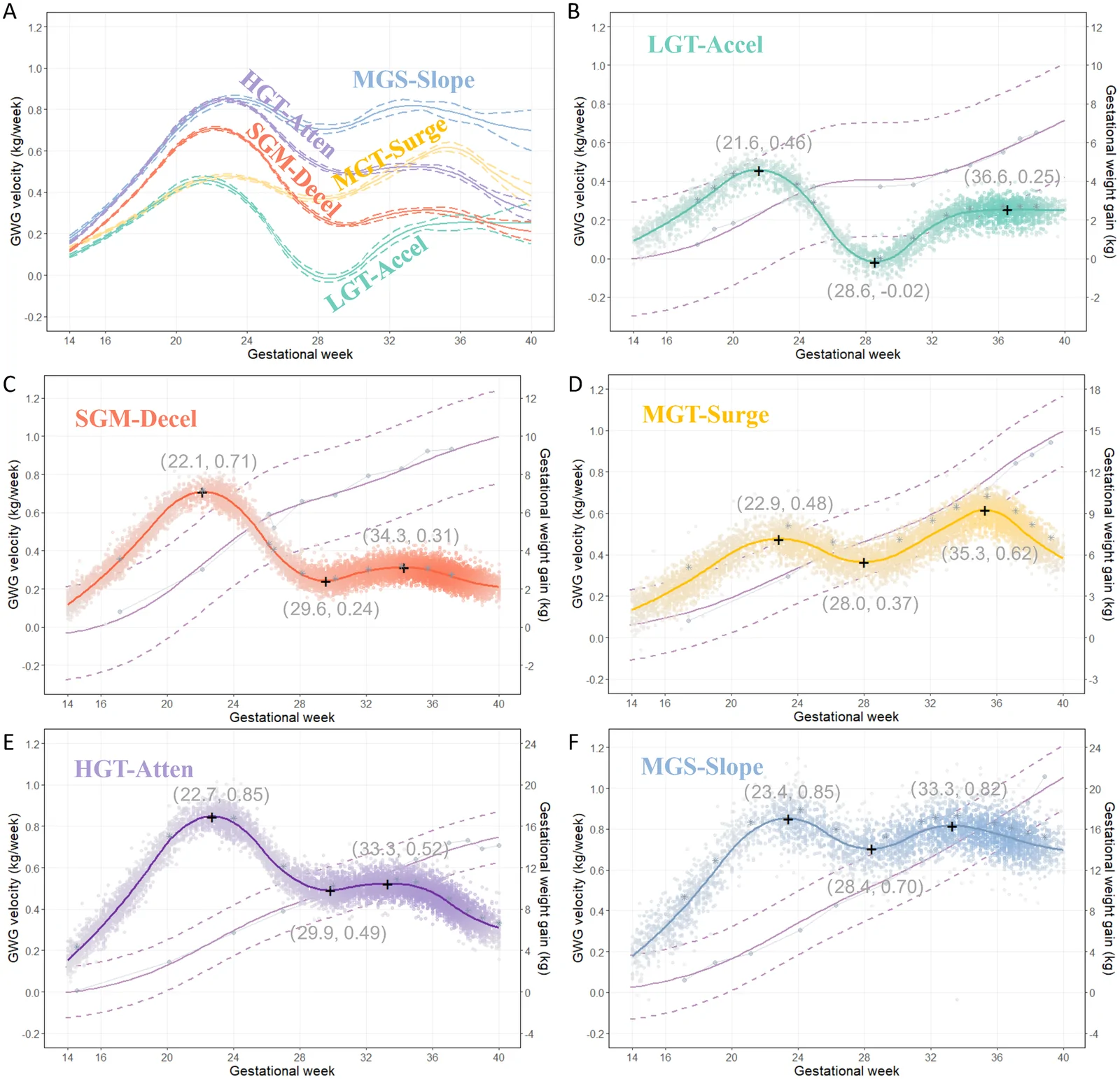
Velocity dynamics of the five gestational weight gain (GWG) trajectory patterns. **(A)** Mean velocity curves (kg/week) for the five identified GWG trajectory groups during the second and third trimesters. **(B–F)** Velocity functions for each specific trajectory group: **(B)** low-gain with terminal acceleration (LGT–Accel), **(C)** steady-gain with mid-gestation deceleration (SGM–Decel) **(D)** modest-gain with terminal surge (MGT–Surge) **(E)** high-gain with trajectory attenuation (HGT–Atten), and **(F)** maximal-gain with steepest slope (MGS–Slope). In each panel, the solid colored line (matched to panel A) denotes the group-level mean velocity trajectory, while scattered points represent predicted velocities of all individuals within the group at their respective observation times. The purple line shows the weight gain trajectory of a representative individual from each group (right *Y*-axis).

We calculated weight gain velocity (in kg/week) using the first derivative of the predicted trajectories from the best LCMM (see Material and Methods). Five distinct GWG trajectory patterns identified in AMA women aligned with the five weight gain velocity curves illustrated in Figure 4A. Although these curves exhibited substantial variations in both velocity magnitude and cumulative weight gain, all shared a consistent four-phase kinetic pattern marked by three inflection points: an initial acceleration phase peaking at approximately 22 weeks; a subsequent deceleration phase reaching its trough near 28 weeks; a re-acceleration phase attaining a second velocity peak at about 35 weeks; and finally, a terminal deceleration phase persisting until delivery.

Among these patterns, the LGT-Accel group exhibited the earliest and lowest initial peak velocity (0.46 kg/week at 21.6 weeks), followed by a pronounced plateau around 28 weeks of gestation where velocity declined to near-zero (−0.02 kg/week at 28.6 weeks), culminating in a markedly attenuated terminal deceleration phase (maintained around 0.25 kg/week post-36.6 weeks, Figure 4B). In contrast, the SGM-Decel group displayed a biphasic deceleration pattern, with velocity peaking early (0.71 kg/week at 22.1 weeks), sharply declining to the deepest trough (0.24 kg/week at 29.6 weeks), and showing only a modest secondary peak (0.31 kg/week at 34.3 weeks, Figure 4C). The MGT-Surge group featured a distinct re-acceleration phase, with velocity rebounding from a trough (0.37 kg/week at 28.0 weeks) to the highest terminal peak (0.62 kg/week at 35.3 weeks), reflecting significant late-gestation momentum (Figure 4D). Meanwhile, the HGT-Atten group demonstrated the highest initial peak velocity (0.85 kg/week at 22.7 weeks) but exhibited progressive attenuation, with a subdued secondary peak (0.52 kg/week at 33.3 weeks) and sustained decline thereafter (Figure 4E). Finally, the MGS-Slope group maintained near-linear acceleration with dual high-magnitude peaks (0.85 kg/week at 23.4 weeks and 0.82 kg/week at 33.3 weeks), minimal mid-gestation decline (trough: 0.70 kg/week at 28.4 weeks), and the highest cumulative gain (20.6 kg, Figure 4F). Both the intra-individual GWG velocity scatter clouds and the representative weight gain trajectories for each group confirmed these velocity patterns (Figures 4B–F and Supplementary Figure 6). In the parity-stratified analyses, the overall trajectory shapes, timing of the major velocity peaks and troughs, and sequence of gestational acceleration and deceleration phases were generally consistent between primiparous and multiparous women (Supplementary Figure 7). Fluctuations were greater in the MGS-Slope and LGT-Accel classes, which included only 74 and 78 primiparous women, respectively.

## 4 Discussion

### 4.1 Main findings

By identifying ranges associated with a relatively low incidence of adverse obstetric outcomes, the CNS formulated recommendations for optimal total GWG and weekly weight gain ranges tailored to Chinese-specific pre-pregnancy BMI categories (28). While clinically pragmatic, these guidelines are primarily based on aggregate measures and provide limited insight into the temporal patterning of weight gain across gestation. As a result, inconsistencies may arise between recommended total GWG and trimester-averaged velocity targets, particularly when longitudinal dynamics are considered (Figure 2A). In our cohort of women with advanced maternal age (AMA), substantial heterogeneity was observed in both total GWG and average weekly gain, with a considerable proportion deviating from guideline-recommended ranges (Figures 2B–D).

Using longitudinal obstetric visit data of AMA women, we identified five distinct GWG trajectory patterns, each exhibiting a consistent four-phase kinetic profile but critical variations in cumulative gain (Figure 3A) and velocity dynamics (Figure 4A). The SGM-Decel group emerged as the optimal reference due to its moderate total GWG (10.3 kg at term) and favorable birth outcomes (Figure 3B). This group demonstrated a biphasic deceleration in weight gain velocity (Figure 4C), marked by an acceleration phase that peaked at 0.71 kg/week around 22 weeks, followed by a rapid decline to a trough of 0.24 kg/week near 30 weeks, a brief mild reacceleration to 0.31 kg/week by approximately 34 weeks, and a final phase of gradual deceleration until delivery.

Conversely, the LGT-Accel and MGT-Surge groups were associated with significantly increased risks of fetal undergrowth outcomes, including low birth weight (LBW) and small for gestational age (SGA). On the other hand, the HGT-Atten and MGS-slope groups were associated with elevated risks of fetal overgrowth phenotypes, such as macrosomia and large for gestational age (LGA). The apparently divergent associations of MGS-Slope with macrosomia, LGA, and LBW should be interpreted in light of the different outcome definitions and the clinical heterogeneity within this trajectory group. LBW is based on an absolute birthweight threshold and may reflect either shortened gestational duration or impaired fetal growth, whereas SGA is standardized for gestational age and fetal sex and more specifically reflects restricted fetal growth. MGS-Slope was not associated with SGA, and its association with LBW was attenuated after additional adjustment for gestational diabetes mellitus and hypertensive disorders of pregnancy. These findings suggest that fetal overgrowth was the more consistent outcome profile of MGS-Slope, whereas the association with LBW was less robust and may partly reflect differences in gestational duration, pregnancy-related clinical conditions, or residual confounding. The sensitivity analysis generally supported the robustness of the principal trajectory–outcome associations.

### 4.2 Interpretation

The biphasic deceleration pattern of the SGM-Decel group is physiologically consistent with prior evidence suggesting that maternal-fetal metabolic demands peak in mid-gestation and decline as placental efficiency stabilizes in late gestation (29). Trajectories characterized by insufficient or unstable weight gain (e.g., LGT-Accel and MGT-Surge) may reflect suboptimal maternal adaptation during pregnancy and were associated with higher risks of fetal growth restriction phenotypes (30, 31). Conversely, trajectories with sustained or excessive gain (HGT-Atten and MGS-Slope) were associated with increased risks of fetal overgrowth outcomes, which may be consistent with maternal metabolic dysregulation and enhanced placental nutrient transfer (32, 33). However, these associations should be interpreted as reflecting differential risk profiles rather than implying causal pathways, particularly in AMA pregnancies where age-related placental and vascular factors play a dominant role in determining pregnancy outcomes.

From a clinical perspective, the proposed modeling framework—integrating conditional growth percentile–based data cleaning with trajectory classification and velocity estimation—provides a dynamic approach to characterizing GWG patterns using routinely collected clinical data. Conventional GWG metrics indicate how much weight has been gained and the average rate of gain over a predefined interval, whereas trajectory and velocity analyses additionally indicate when and how that gain occurred. This temporal information may help distinguish women with similar cumulative GWG but different phase-specific patterns. For example, limited mid-gestational gain or marked deceleration may prompt closer assessment of fetal growth, whereas persistently high or rapidly accelerating gain may warrant closer monitoring of excessive maternal gain and fetal size. These trajectory patterns are not intended to serve as prescriptive intervention thresholds; rather, they may complement existing guideline-based care by supporting more refined risk characterization and phase-specific antenatal monitoring.

### 4.3 Strengths and limitations

This work provides key methodological advantages in GWG monitoring. First, the CGP approach enables high-throughput screening of longitudinal trajectories and rigorous identification and exclusion of implausible measurements, thereby preventing both excessive data removal and false retention that occur when population distribution thresholds are used for outlier detection (18). Second, we established a flexible modeling framework to characterize the time-varying rate of gestational weight gain, allowing both cumulative gain and phase-specific velocity dynamics to be quantified. This framework revealed a consistent four-phase kinetic structure across trajectories and provided a more nuanced depiction of GWG dynamics than aggregate measures alone (34).

Several limitations should also be acknowledged. As with all retrospective cohort studies, missing data and irregular follow-up were unavoidable. Despite adjusting for major pregnancy complications such as gestational diabetes and hypertensive disorders, the inability to account for smoking and socioeconomic status represents a source of residual confounding that should be acknowledged. In addition, while trajectory-level associations with obstetric outcomes were examined, we did not assess stage-specific velocity–outcome relationships, which limits the ability to derive precise, phase-targeted weight management recommendations. Finally, given the observational design, the identified GWG patterns should be viewed as dynamic risk indicators rather than causal determinants of adverse outcomes.

## 5 Conclusion

Our findings show that gestational weight gain in AMA pregnancies follows heterogeneous trajectory and velocity patterns that are differentially associated with fetal growth–related outcomes. By characterizing GWG dynamics rather than relying solely on total gain, our findings highlight the potential value of trajectory-based approaches for early risk stratification and individualized antenatal monitoring in advanced maternal age pregnancies, without replacing existing guideline-based clinical management.

## Data Availability

The raw data supporting the conclusions of this article will be made available by the authors, without undue reservation.
