# Supplementary material for: Modeling dynamic velocity-based gestational weight gain trajectories in advanced maternal age: association with perinatal outcomes
Source: Front Med (Lausanne). 2026 Sep 9;13:1782650. doi: 10.3389/fmed.2026.1782650 (PMC13598080; doi:10.3389/fmed.2026.1782650)
Supplement: Supplementary file 1 [file Supplementaryfile1.docx]

Supplementary Material

# 1 Supplementary Figures and Tables

## 1.1 Supplementary Figures

**Supplementary Figure 1.** **Data quality assessment and preprocessing of gestational weight measurements.** **(A)** Number of gestational weight observations at each gestational week. The black smooth curve represents the kernel density estimate. **(B)** Frequency of weight measurements among pregnant women; the blue line indicates the cumulative probability, with the red dashed horizontal line marking the 50% cumulative probability. **(C)** Individual gestational weight change trajectories for all participants. **(D)** Individual gestational weight change trajectories after outlier removal. Gray lines are the original trajectories from panel C; red points indicate outliers identified by conditional growth percentiles. After removing these outliers, the remaining data points are connected by black lines.

**Supplementary Figure 2.** **Determination of gestational weight gain (GWG). (A)** Frequency distribution of gestational age at the first measurement. **(B)** Comparison of true weight and predicted weight during the first trimester using the aforementioned mixed-effect model, indicating a justifiable range of predictive error.

**Supplementary Figure 3.** **Selection of the optimal latent class mixed model. (A)** Bayesian information criterion values corresponding to different numbers of latent classes and degrees of freedom for natural cubic splines. **(B)** Proportion of participants in each group for models with 6 degrees of freedom across different numbers of latent classes; groups with proportions below 10% are marked with red points. **(C)** Mean posterior probability of each group for models with 6 degrees of freedom across different numbers of latent classes. **(D)** Proportion of participants with posterior probabilities greater than 70% in each group for models with 6 degrees of freedom across different numbers of latent classes; groups with proportions below 65% are marked with red points.

**Supplementary Figure 4. Gestational weight trajectories of participants across the four pre-pregnancy BMI categories during the second and third trimesters.** **(A)** Underweight, **(B)** Normal weight, **(C)** Overweight, **(D)** Obese. The green lines are the reference curves for mid-late pregnancy GWG progression relative to weight gain at 13 weeks according to the CNS guidelines.

**Supplementary Figure 5.** **Five patterns of gestational weight gain (GWG) trajectories identified by the latent class mixed model.** They are LGT-Accel (Low-Gain with Terminal Acceleration), SGM-Decel (Steady-Gain with Mid-Gestation Deceleration), MGT-Surge (Modest-Gain with Terminal Surge), HGT-Atten (High-Gain with Trajectory Attenuation), and MGS-Slope (Maximal-Gain with Steepest Slope). Gray solid lines represent individual weight gain trajectories. Blue solid lines are the average weight gain trajectory, and blue dashed lines indicate the 95% confidence band.

**Supplementary Figure 6.** **Representative individuals from the five GWG trajectory patterns.** (I) LGT-Accel, (II) SGM-Decel, (III) MGT-Surge, (IV) HGT-Atten, and (V) MGS-Slope. For each pattern, five individuals are shown. Gray lines represent the observed individual weight gain trajectories; red solid lines represent the corresponding trajectories fitted by the latent class mixed model; red dashed lines indicate the ±4 standard deviation intervals around the fitted trajectories. (LGT-Accel: Low-Gain with Terminal Acceleration; SGM-Decel: Steady-Gain with Mid-Gestation Deceleration; MGT-Surge: Modest-Gain with Terminal Surge; HGT-Atten: High-Gain with Trajectory Attenuation; MGS-Slope: Maximal-Gain with Steepest Slope)

**Supplementary Figure 7. Parity-stratified gestational weight gain trajectories and velocity patterns within the five trajectory classes.** Panels A, C, E, G, and I show the fitted gestational weight gain trajectories for the MGS-Slope, MGT-Surge, LGT-Accel, SGM-Decel, and HGT-Atten classes, respectively. Panels B, D, F, H, and J show the corresponding weight gain velocity curves, obtained as the first derivatives of the fitted trajectories. Curves are presented for the original class-specific model fitted to all women (“Overall”), primiparous women (“Parity = 1”), and multiparous women (“Parity > 1”).

## 1.2 Supplementary Tables

**Supplementary Table 1. Maternal and infants baseline characteristics by pre-pregnancy BMI groups.**

| **Charateristics** | | | **Overall** | **Pre-pregnancy BMI** | | | | ***P* value** |
| --- | --- | --- | --- | --- | --- | --- | --- | --- |
|  |  |  |  | **Underweight** | **Normal weight** | **Overweight** | **Obesity** |  |
| Total | |  | 3,818  (100.0) | 222  (5.8) | 2,420  (63.4) | 926  (24.3) | 250  (6.6) |  |
| **Maternal characteristics** | | | |  |  |  |  |  |
|  | Age (years) | |  |  |  |  |  | *<0.001* |
|  |  | Mean ± SD | 38.0 ±  2.4 | 37.5 ±  2.1 | 37.9 ±  2.3 | 38.4 ±  2.5 | 37.9 ±  2.4 |  |
|  | Age (years), n (%) | | |  |  |  |  | *<0.001* |
|  |  | Advanced age: 35~40 | 3,065 (80.3) | 189  (85.1) | 1,980  (81.8) | 694  (74.9) | 202  (80.8) |  |
|  |  | Super-advanced age: ≥ 40 | 753 (19.7) | 33  (14.9) | 440  (18.2) | 232  (25.1) | 48  (19.2) |  |
|  | Delivery gestations (weeks) | | |  |  |  |  | *<0.001* |
|  |  | Mean ± SD | 38.4 ±  1.7 | 38.4 ±  1.4 | 38.4 ±  1.6 | 38.2 ±  1.8 | 37.9 ±  2.0 |  |
|  | Parity (times), n (%) | | |  |  |  |  | *0.299* |
|  |  | 1 | 655  (17.2) | 48  (21.6) | 403  (16.7) | 159  (17.2) | 45  (18.0) |  |
|  |  | ≥2 | 3,163  (82.8) | 174  (78.4) | 2,017  (83.3) | 767  (82.8) | 205  (82.0) |  |
|  | Mode of conception, n (%) | | | |  |  |  | *0.040* |
|  |  | Natural conception | 3,354  (87.8) | 203  (91.4) | 2,141  (88.5) | 792  (85.5) | 218  (87.2) |  |
|  |  | Assisted reproductive technology, ART | 464  (12.2) | 19  (8.6) | 279  (11.5) | 134  (14.5) | 32  (12.8) |  |
|  | Mode of delivery, n (%) | | |  |  |  |  | *<0.001* |
|  |  | Natural labour | 1,333  (34.9) | 105  (47.3) | 910  (37.6) | 269  (29.0) | 49  (19.6) |  |
|  |  | Cesarean section | 2,485  (65.1) | 117  (52.7) | 1,510  (62.4) | 657  (71.0) | 201  (80.4) |  |
|  | Total GWG (kg) | | |  |  |  |  | *<0.001* |
|  |  | Mean ± SD | 10.7 ± 3.7 | 11.6 ±  3.5 | 11.1 ±  3.5 | 9.9 ±  3.8 | 8.4 ±  3.9 |  |
|  | Total GWG by CNS recommendation, n (%) | | | | |  |  | *<0.001* |
|  |  | Insufficient | 795 (20.8) | 102  (45.9) | 440  (18.2) | 204  (22.0) | 49  (19.6) |  |
|  |  | Adequate | 2,119  (55.5) | 101  (45.5) | 1,530  (63.2) | 381  (41.1) | 107  (42.8) |  |
|  |  | Excessive | 904  (23.7) | 19  (8.6) | 450  (18.6) | 341  (36.8) | 94  (37.6) |  |
| Infant charateristics | | | |  |  |  |  |  |
|  | Sex | |  |  |  |  |  | *0.680* |
|  |  | Boys | 2,150  (56.3) | 121  (54.5) | 1,358  (56.1) | 535  (57.8) | 136 (54.4) |  |
|  |  | Girls | 1,668  (43.7) | 101  (45.5) | 1,062  (43.9) | 391  (42.2) | 114 (45.6) |  |
|  | Pre-term birth, n (%) | | |  |  |  |  | *<0.001* |
|  |  | Yes | 431  (11.3) | 25  (11.3) | 241  (10.0) | 118  (12.7) | 47  (18.8) |  |
|  |  | No | 3,387  (88.7) | 197  (88.7) | 2179  (90.0) | 808  (87.3) | 203  (81.2) |  |
|  | Low birth weight, n (%) | | |  |  |  |  | *0.447* |
|  |  | Yes | 330  (8.6) | 24  (10.8) | 204  (8.4) | 76  (8.2) | 26  (10.4) |  |
|  |  | No | 3,488  (91.4) | 198  (89.2) | 2,216  (91.6) | 850  (91.8) | 224  (89.6) |  |
|  | Intrauterine growth, n (%) | | | |  |  |  | *<0.001* |
|  |  | SGA | 297  (7.8) | 29  (13.1) | 196  (8.1) | 52  (5.6) | 20  (8.0) |  |
|  |  | AGA | 3,027  (79.3) | 178  (80.2) | 1,958  (80.9) | 719  (77.6) | 172  (68.8) |  |
|  |  | LGA | 494  (12.9) | 15  (6.8) | 266  (11.0) | 155  (16.7) | 58  (23.2) |  |

Note: Data are presented as mean ± standard deviation (SD) for continuous variables and n (%) for categorical variables. P-values were derived from analysis of variance (ANOVA) for continuous variables and χ² tests for categorical variables. Abbreviations: GWG, gestational weight gain; CNS, Chinese Nutrition Society; SGA, small for gestational age; AGA, appropriate for gestational age; LGA, large for gestational age.

**Supplementary Table 2. Maternal and infants characteristics by GWG trajectory patterns.**

| **Charateristics** | | | **Overall** | **Trajectory patterns** | | | | | ***P* value** |
| --- | --- | --- | --- | --- | --- | --- | --- | --- | --- |
|  |  |  |  | **LGT-Accel** | **SGM-Decel** | **MGT-Surge** | **HGT-Atten** | **MGS-Slope** |  |
| Total |  |  | 3,818 (100.0) | 404 (10.6) | 1,133 (29.7) | 825 (21.6) | 1,014 (26.6) | 442 (11.6) |  |
| **Maternal characteristics** | | | |  |  |  |  |  |  |
|  | Age (years) | | |  |  |  |  |  | *<0.001* |
|  |  | Mean ± SD | 38.0 ± 2.4 | 38.5 ± 2.7 | 38.1 ± 2.4 | 38.1 ± 2.5 | 37.9 ± 2.3 | 37.5 ± 2.1 |  |
|  | Age (years), n (%) | | |  |  |  |  |  | *<0.001* |
|  |  | Advanced age: 35~40 | 3,065 (80.3) | 285 (70.5) | 913 (80.6) | 647 (78.4) | 837 (82.5) | 383 (86.7) |  |
|  |  | Super-advanced age: ≥ 40 | 753 (19.7) | 119 (29.5) | 220 (19.4) | 178 (21.6) | 177 (17.5) | 59 (13.3) |  |
|  | Pre-pregnant BMI (kg/m^2^) | | | |  |  |  |  | *<0.001* |
|  |  | Mean ± SD | 22.8 ± 3.2 | 25.1 ± 3.6 | 22.7 ± 3.0 | 22.9 ± 3.3 | 22.1 ± 2.8 | 22.4 ± 2.8 |  |
|  | Category for pre-pregnant BMI, n (%) | | | | |  |  |  | *<0.001* |
|  |  | Underweight | 222  (5.8) | 7  (1.7) | 62  (5.5) | 52  (6.3) | 68  (6.7) | 33  (7.5) |  |
|  |  | Normal weight | 2,420  (63.4) | 165  (40.8) | 727  (64.2) | 505  (61.2) | 720  (71.0) | 303  (68.6) |  |
|  |  | Overweight | 926  (24.3) | 158  (39.1) | 282  (24.9) | 204  (24.7) | 195  (19.2) | 87  (19.7) |  |
|  |  | Obesity | 250  (6.5) | 74  (18.3) | 62  (5.5) | 64  (7.8) | 31  (3.1) | 19  (4.3) |  |
|  | Delivery gestations (weeks) | | | |  |  |  |  | *0.002* |
|  |  | Mean ± SD | 38.4 ± 1.7 | 38.3 ± 1.5 | 38.5 ± 1.6 | 38.2 ± 1.8 | 38.4 ± 1.7 | 38.2 ± 1.7 |  |
|  | Parity (times), n (%) | | | |  |  |  |  | *0.673* |
|  |  | 1 | 655 (17.2) | 78 (19.3) | 184 (16.2) | 139 (16.8) | 180 (17.8) | 74 (16.7) |  |
|  |  | ≥2 | 3,163 (82.8) | 326 (80.7) | 949 (83.8) | 686 (83.2) | 834 (82.2) | 368 (83.3) |  |
|  | Mode of conception, n (%) | | | |  |  |  |  | *0.198* |
|  |  | Natural conception | 3,354 (87.8) | 347 (85.9) | 980 (86.5) | 729 (88.4) | 902 (89.0) | 396 (89.6) |  |
|  |  | Assisted reproductive technology, ART | 464 (12.2) | 57 (14.1) | 153 (13.5) | 96 (11.6) | 112 (11.0) | 46 (10.4) |  |
|  | Mode of delivery, n (%) | | | |  |  |  |  | *<0.001* |
|  |  | Natural labour | 1,333 (34.9) | 142 (35.1) | 457 (40.3) | 286 (34.7) | 324 (32.0) | 124 (28.1) |  |
|  |  | Cesarean section | 2,485 (65.1) | 262 (64.9) | 676 (59.7) | 539 (65.3) | 690 (68.0) | 318 (71.9) |  |
|  | Total GWG (kg) | | |  |  |  |  |  | *<0.001* |
|  |  | Mean ± SD | 10.7 ± 3.7 | 5.2 ± 1.8 | 8.9 ± 1.7 | 10.1 ± 2.5 | 12.8 ± 2.0 | 16.3 ± 3.1 |  |
|  | Total GWG by CNS recommendation, n (%) | | | | | |  |  | *<0.001* |
|  |  | Insufficient | 795 (20.8) | 339 (83.9) | 288 (25.4) | 138 (16.7) | 26 (2.6) | 4 (0.9) |  |
|  |  | Adequate | 2,119 (55.5) | 64 (15.8) | 794 (70.1) | 574 (69.6) | 608 (60.0) | 79 (17.9) |  |
|  |  | Excessive | 904 (23.7) | 1 (0.2) | 51 (4.5) | 113 (13.7) | 380 (37.5) | 359 (81.2) |  |
| **Infant charateristics** | | | |  |  |  |  |  |  |
|  | Sex | |  |  |  |  |  |  | *0.040* |
|  |  | Boys | 2,150 (56.3) | 221 (54.7) | 635 (56.0) | 472 (57.2) | 599 (59.1) | 223 (50.5) |  |
|  |  | Girls | 1,668 (43.7) | 183 (45.3) | 498 (44.0) | 353 (42.8) | 415 (40.9) | 219 (49.5) |  |
|  | Pre-term birth, n (%) | | |  |  |  |  |  | *0.072* |
|  |  | Yes | 431 (11.3) | 45 (11.1) | 118 (10.4) | 109 (13.2) | 99 (9.8) | 60 (13.6) |  |
|  |  | No | 3,387 (88.7) | 359 (88.9) | 1015 (89.6) | 716 (86.8) | 915 (90.2) | 382 (86.4) |  |
|  | Low birth weight, n (%) | | | |  |  |  |  | *<0.001* |
|  |  | Low | 330 (8.6) | 39 (9.7) | 71 (6.3) | 99 (12.0) | 75 (7.4) | 46 (10.4) |  |
|  |  | Normal | 3,389 (88.8) | 358 (88.6) | 1,044 (92.1) | 711 (86.2) | 895 (88.3) | 381 (86.2) |  |
|  |  | Macrosomia | 99 (2.6) | 7 (1.7) | 18 (1.6) | 15 (1.8) | 44 (4.3) | 15 (3.4) |  |
|  | Intrauterine growth, n (%) | | | |  |  |  |  | *<0.001* |
|  |  | SGA | 297 (7.8) | 38 (9.4) | 75 (6.6) | 90 (10.9) | 59 (5.8) | 35 (7.9) |  |
|  |  | AGA | 3,027 (79.3) | 317 (78.5) | 932 (82.3) | 657 (79.6) | 790 (77.9) | 331 (74.9) |  |
|  |  | LGA | 494 (12.9) | 49 (12.1) | 126 (11.1) | 78 (9.5) | 165 (16.3) | 76 (17.2) |  |

Note: Data are presented as mean ± standard deviation (SD) for continuous variables and n (%) for categorical variables. P-values were derived from analysis of variance (ANOVA) for continuous variables and χ² tests for categorical variables. GWG trajectory patterns: LGT-Accel (Low-Gain with Terminal Acceleration), SGM-Decel (Steady-Gain with Mid-Gestation Deceleration), MGT-Surge (Modest-Gain with Terminal Surge), HGT-Atten (High-Gain with Trajectory Attenuation), MGS-Slope (Maximal-Gain with Steepest Slope). Other abbreviations: BMI, body mass index; GWG, gestational weight gain; CNS, Chinese Nutrition Society; SGA, small for gestational age; AGA, appropriate for gestational age; LGA, large for gestational age.

**Supplementary Table 3. Associations between gestational weight gain trajectory groups and perinatal outcomes under alternative adjustment models.**

| **Outcome** | **Trajectory group** | **Model 1** | | **Model 2** | |
| --- | --- | --- | --- | --- | --- |
|  |  | **OR (95% CI)** | **P value** | **OR (95% CI)** | **P value** |
| **PTB** | SGM-Decel | Reference |  | Reference |  |
|  | LGT-Accel | 0.89 (0.61-1.29) | 0.552 | 0.87 (0.59-1.27) | 0.474 |
|  | MGT-Surge | 1.27 (0.96-1.68) | 0.092 | 1.11 (0.83-1.48) | 0.481 |
|  | HGT-Atten | 0.95 (0.72-1.27) | 0.740 | 0.90 (0.67-1.20) | 0.465 |
|  | MGS-Slope | 1.37 (0.98-1.91) | 0.065 | 1.14 (0.80-1.62) | 0.472 |
| **LBW** | SGM-Decel | Reference |  | Reference |  |
|  | LGT-Accel | **1.55 (1.02**-**2.36)** | **0.040** | 1.51 (0.98-2.33) | 0.059 |
|  | MGT-Surge | **2.01 (1.46**-**2.78)** | **<0.001** | **1.69 (1.21**-**2.35)** | **0.002** |
|  | HGT-Atten | 1.19 (0.84-1.67) | 0.326 | 1.08 (0.76-1.53) | 0.682 |
|  | MGS-Slope | **1.65 (1.12**-**2.45)** | **0.012** | 1.24 (0.82-1.89) | 0.310 |
| **Macrosomia** | SGM-Decel | Reference |  | Reference |  |
|  | LGT-Accel | 0.80 (0.33-1.98) | 0.634 | 0.79 (0.32-1.95) | 0.603 |
|  | MGT-Surge | 1.17 (0.58-2.34) | 0.661 | 1.25 (0.62-2.51) | 0.536 |
|  | HGT-Atten | **3.12 (1.78**-**5.48)** | **<0.001** | **3.22 (1.82**-**5.71)** | **<0.001** |
|  | MGS-Slope | **2.45 (1.21**-**4.95)** | **0.013** | **2.67 (1.30**-**5.52)** | **0.008** |
| **SGA** | SGM-Decel | Reference |  | Reference |  |
|  | LGT-Accel | **1.61 (1.06**-**2.46)** | **0.027** | **1.61 (1.04**-**2.47)** | **0.031** |
|  | MGT-Surge | **1.69 (1.22**-**2.34)** | **0.002** | **1.51 (1.08**-**2.11)** | **0.015** |
|  | HGT-Atten | 0.85 (0.60-1.22) | 0.391 | 0.80 (0.55-1.15) | 0.226 |
|  | MGS-Slope | 1.28 (0.83-1.96) | 0.261 | 1.08 (0.69-1.67) | 0.747 |
| **LGA** | SGM-Decel | Reference |  | Reference |  |
|  | LGT-Accel | 0.87 (0.60-1.26) | 0.468 | 0.83 (0.57-1.21) | 0.334 |
|  | MGT-Surge | 0.83 (0.61-1.12) | 0.226 | 0.88 (0.65-1.20) | 0.420 |
|  | HGT-Atten | **1.64 (1.27**-**2.12)** | **<0.001** | **1.74 (1.34**-**2.27)** | **<0.001** |
|  | MGS-Slope | **1.71 (1.24**-**2.35)** | **<0.001** | **1.92 (1.38**-**2.66)** | **<0.001** |

Note: The SGM-Decel group was used as the reference group in all analyses. Model 1 was adjusted for maternal age, pre-pregnancy body mass index, mode of conception, parity, and fetal sex. Model 2 was additionally adjusted for gestational diabetes mellitus and hypertensive disorders of pregnancy. Statistically significant results are shown in bold. Red font highlights associations that were attenuated after additional adjustment.
